# Positive effects on activities of daily living one year after receiving comprehensive geriatric assessment – results from the randomised controlled study CGA-Swed

**DOI:** 10.1186/s12877-022-02862-6

**Published:** 2022-03-03

**Authors:** Katarina Wilhelmson, Isabelle Andersson Hammar, Theresa Westgård, Lena Holmquist Henrikson, Synneve Dahlin-Ivanoff

**Affiliations:** 1 Department of Health and Rehabilitation, Institute of Neuroscience and Physiology, The Sahlgrenska Academy, University of Gothenburg, Box 455, 405 30 Gothenburg, Sweden; 2 Centre of Aging and Health-AGECAP, University of Gothenburg, Gothenburg, Sweden; 3 Department of Acute Medicine and Geriatrics, The Sahlgrenska University Hospital, Region Västra Götaland, 413 45 Gothenburg, Sweden; 4 The Geriatric Development Centre, The Sahlgrenska University Hospital, Region Västra Götaland, 413 45 Gothenburg, Sweden; 5 Department of Psychiatry and Neurochemistry, Institute of Neuroscience and Physiology, The Sahlgrenska Academy, University of Gothenburg, Gothenburg, Sweden

**Keywords:** Frailty, Intervention, Hospital care, Activities of daily living, Comprehensive geriatric assessment

## Abstract

**Background:**

Today’s acute hospital care is poorly adapted to the complex needs of frail older people. This exposes them to avoidable risks, such as loss of functional capacities, leading to unnecessary health and social care needs. Being frail and in need of acute hospital care often leads to higher dependence in Activities of Daily Living (ADL), especially if one’s needs are not acknowledged. Comprehensive Geriatric Assessment (CGA) is one way to meet frail older people’s complex needs. The study’s aim was to investigate the effects on frail older people’s ADL 12 months after receiving CGA.

**Methods:**

This is a two-armed randomised controlled intervention study. Participants were frail older people (75+) who sought the emergency department and needed admission to a medical ward. The intervention was CGA performed at a geriatric management unit during the hospital stay. The CGA included comprehensive assessment of medical, functional, psychological, social, and environmental status as well as treatment, rehabilitation, discharge planning, and follow-up. Multidisciplinary teamwork and a person-centred approach were used. The control was care at an ordinary medical hospital ward. The primary outcome was change in dependence in ADL from 2 weeks before admission to the 12-month follow-up.

**Results:**

At admission, 155 people participated (77 in the control, 78 in the intervention). At the 12-month follow-up, 78 participated (40 in the control, 38 in the intervention). Attrition was mainly due to mortality. Four participants in the control (5.2%) and twelve in the intervention group (15.4%) had improved in their ADL 1 year after discharge (OR = 3.32; 95% CI = 1.02–10.79).

**Conclusions:**

In-hospital CGA performed at a geriatric management unit improves frail older people’s ADL. Being less dependent in ADL increases frail older people’s ability to remain in their own housing, which is important for both the individual and society.

**Trial registration:**

ClinicalTrials.gov, NCT02773914. Retrospectively registered 16 May 2016.

**Supplementary Information:**

The online version contains supplementary material available at 10.1186/s12877-022-02862-6.

## Background

Frail older people are at high risk of developing chronic diseases and dependence in activities of daily living (ADL) [1, 2], leading to the need of both social and health care. However, their needs are often not recognised because health care lacks knowledge about frail older people and how to best assess and meet their complex needs [3, 4]. Being frail and in need of acute hospital care often leads to higher dependence in ADL, especially if one’s needs are not acknowledged [5]. After an acute hospital stay, frail older people are at high risk of not regaining their pre-hospital ADL status, leading to increased dependence in ADL [6]. Despite much evidence that frail older people benefit from care according to Comprehensive Geriatric Assessment (CGA), there are still substantial gaps within health and social care regarding this method of taking care of frail older people [7–10]. CGA remains a rather unknown concept within Swedish hospital care [7]. Thus, interventions and evaluations of how to best meet frail older people’s needs when requiring acute hospital care are needed. In 2016, the CGA-Swed study [11] started. It is a two-armed randomised controlled study that aims to evaluate the effects of CGA for frail older people in Swedish acute hospital settings, with changes in dependence in ADL as the primary outcome.

Many studies and reviews have shown benefits of comprehensive geriatric assessment for frail older people during hospital stays. The benefits have been shown for functional status, readmissions, and ability to remain in one’s own housing [7–10]. These outcomes are important both for the person and for society as they affect the individual’s quality of life and impact on the cost of care services for society. CGA is performed by a multidisciplinary team and includes systematic comprehensive team assessment and treatment. It focuses on determining the medical, functional, mental, and social capabilities of frail older people [9]. It uses a person-centred approach [12] and takes the frail older person’s own resources into account [13]. The goal of CGA is to increase the frail older person’s quality of life and ADL [2], even though many of the chronic diseases that frail older people suffer from cannot be cured.

The effects on ADL have been inconclusive [9, 10]. Being dependent in ADL has great impact on the ability to remain living in one’s own housing [14]. The home is an important arena for older people [15]. It has been pointed out that it is important to focus on prevention of dependence in ADL in clinical care for frail older people [2]. Cochrane reviews showed that more older people were able to be discharged back to their own housing after receiving CGA as compared to those who did not [9, 10]. However, the reviews did not identify any significant effects on ADL status after having received CGA [9, 10]. This is somewhat surprising since the level of dependence in ADL influences the possibility of remaining in one’s own housing [14]. The CGA-Swed study also failed to show significant effect on ADL in the short term, up to 6 months [16], but the participants were also followed up after 12 months. The primary outcome of the CGA-Swed study was the long-term change in ADL at the 12-month follow-up [11]. Thus, the aim was to investigate the long-term effects on frail older people’s ADL 12 months after receiving CGA in an acute hospital care setting in Sweden.

## Methods

### Study design

The CGA-Swed is a two-armed randomised controlled study performed at Sahlgrenska University Hospital in Gothenburg, Sweden. A pilot and feasibility study [17] was conducted first. No major changes were made after the study commenced. The CGA-Swed has also been described in the study protocol [11]. The study is approved by the Regional Ethical Review Board in Gothenburg, ref. no: 4899–15. Trial Registration: ClinicalTrials.gov, NCT02773914.

### Participants

To be included in the study, participants had to be 75 years or older, in need of admission to a medicine or geriatric ward, and screened as frail according to the FRESH-screening [11, 18]. Exclusion criteria were being in need of a higher level of care than the study wards could provide (such as needing e.g. telemetry or intensive care), and not being admitted through the emergency department (due to a ‘fast track’ for predefined diagnoses such as stroke, acute myocardial infarction, and hip fracture). Participants were invited to participate by the hospital bed coordinator at the emergency department. Those who agreed to participate signed a consent form. Some of the participants had cognitive impairments that made them unable to understand the information well enough to give their consent. In these cases, their next of kin signed the consent form.

### Randomisation and blinding

The participants were allocated to the intervention or to the control group by the hospital bed coordinator, with a one-to-one ratio. The randomisation was done using sealed opaque envelopes with computer-generated numbers created by one of the researchers using QuickCalcs at Graphpad (https://graphpad.com/quickcalcs/randomN1.cfm). The hospital bed coordinator had no further involvement in the study.

The researchers who conducted the interviews during hospital stay, the ward staff, and the participants could not be blinded to allocation. The follow-ups were carried out by the same researcher who had conducted the first interview, leading to a risk of the researcher not being blinded to allocation at the follow-up. However, due to the large number of interviews performed by the same person during the hospital stay, the allocation was not usually recalled. If the researcher doing the follow-up was aware of the allocation, or if the participant revealed it during the interview, the researcher stated this in the questionnaire. This occurred in only one documented case. Thus, the researchers doing the follow-up interviews were in almost all cases blinded to allocation.

### Procedures

The intervention was CGA performed at a geriatric management unit. The CGA included comprehensive assessment of medical, functional, psychological, social, and environmental status as well as treatment, rehabilitation, discharge planning, and follow-up [11]. It was performed by a multidisciplinary team. The team consisted of a geriatrician, a registered nurse, an assistant nurse, a physiotherapist, and an occupational therapist, and when needed also a social worker and a dietician. The multidisciplinary teamwork was person-centred. A team conference was held every weekday, where the team shared information and used their experience and competence to tailor the care to the needs of each frail older patient. During the discharge planning, the CGA-ward could recommend follow-ups to be carried out by primary care and/or social care providers. However, it was not possible for the CGA-ward to check whether the primary or social care providers acknowledged and implemented such recommendations. The geriatric clinic itself did not follow up on the discharged patients. For further details, see the study protocol article [11] and the pilot and feasibility study [17].

The control group received care at an ordinary medical ward at the same hospital. At this ward, the staff did not work according to CGA and did not use a specialised multi-disciplinary team. The control ward had access to a physiotherapist and occupational therapist on a when-in-need basis. There were no geriatricians at the control ward.

### Data collection

We interviewed the participants during their hospital stay, within a couple of days after they had been admitted, using a structured questionnaire. Some participants were discharged before the interview could be performed. In these cases, the interviews were conducted at their home. The interviews were complemented with a chart review of information during the hospital stay. Follow-ups were performed at 1, 6 and 12 months after discharge, with an interview in the participant’s home. Participants who did not want to be visited at home took part in a shorter interview by phone instead.

### Outcomes

*The primary outcome* was change in dependence in ADL by the 12-month follow-up, from 2 weeks before admission and from the point of admission. Two weeks prior to admission ought to better reflect the person’s habitual status, since being acutely ill often leads to higher ADL dependence. This was the reason for using ADL dependence 2 weeks prior to hospital admission, and not merely ADL dependence during admission. ADL were measured using the ADL-staircase [19], which includes dependence in four instrumental ADL (IADL): cleaning, shopping, cooking, and transportation, and five personal ADL (PADL): bathing, dressing, going to the toilet, transferring, and feeding – 9 activities in total. Continence was omitted since it was not considered to be an activity. If another person was involved in the activity by giving personal or directive assistance, the participant was considered to be dependent in the activity. The sum of ADL dependence was calculated, range 0–9, and a change of ≥1 unit – that is, being dependent in one more or one less activity of daily living – was deemed a clinically significant change.

*Secondary outcomes* were change in self-rated health and frailty, from admission to the 12-month follow-up. Self-rated health was measured by asking: ‘In general, would you say your health is’, with the response alternatives: ‘excellent, very good, good, fair, and poor’. A change of ≥1 unit was deemed a clinically significant change. The following eight frailty indicators were used [20, 21]: 1) Fatigue: Answering yes to the question ‘Have you suffered any general fatigue/tiredness over the last three months?’; 2) Weight loss: Answering yes to the question ‘Have you suffered any weight loss over the last three months?’; 3) Reduced physical activity: Taking 1–2 or fewer outdoor walks per week; 4) Weakness: Grip strength measured with a North Coast dynamometer, with reduced strength considered to be below the lowest norm range for ages 80–84, 13 kg for women and 21 kg for men for the dominant hand, and 10 kg for women and 18 kg form men for the non-dominant hand [22]; 5) Reduced gait speed: Walking four metres with a speed of ≤0.6 m/second [23]; 6) Visual impairment: Having a visual acuity of 0.5 or less on the KM chart [24]; 7) Impaired cognition: Having a score below 25 on the Mini-Mental State Examination [25]; and 8) Impaired balance: Having a value of 47 or less on the Berg Balance Scale [26]. The sum of the frailty indicators was calculated, range 0–8, and a change of ≥1 unit was deemed a clinically significant change.

### Statistical analysis

The base for the power calculation was the primary outcome, dependence in ADL (range 0–9), with an assumed difference between the intervention and control groups of one dependence and a standard deviation of two in both groups. At least 64 participants were needed in each group to detect a difference between the intervention and control groups with a two-sided test and with a significance level of α = 0.05 and 80% power. As there were high risks of loss to follow-up, we planned to include 156 participants in total, 78 in each group. The power calculation and the assumed loss to follow-up were based on previous research on similar populations [27].

The analyses were done on complete cases, i.e. those participating both at admission and at the 12-month follow-up, and on the basis of the intention-to-treat principle (ITT), i.e. on all participants based on the group they were randomised to and irrespective of the extent to which CGA was received. However, a rather high dropout rate was inevitable as the participants were frail older people in need of acute hospital care. For the ITT analysis, we imputed data to replace missing values based on the median change of deterioration between 2 weeks prior to admission/during hospital stay and the follow-up [27]. We chose this method for data imputation because the study sample was expected to deteriorate over time and deteriorated health is often the reason for not participating in follow-ups. Worst case change was imputed for those who had died before the follow-up (that is, they were given the worst possible estimate at the follow-up).

In addition, a subgroup analysis was done without nursing home residents (five in the control group, and seven in the intervention group). This was done because the occupational therapist did not prioritise nursing home residents. Thus, most of these participants were not assessed by an occupational therapist, even though this assessment is an important part of the CGA.

To test the difference in the proportions between the two groups, we used the chi-square test. To analyse change over time, we calculated the number of participants that had improved, maintained, or decreased their level of ADL dependence, self-rated health, and frailty at the 12-month follow-up compared to 2 weeks before admission and/or at admission. Odds ratios (OR) were used to compare the intervention group with the control group, with the control group as the reference group. For all analyses, we used two-sided significance tests, with a value of *p* < 0.05 and a 95% confidence interval (CI) considered as statistically significant. The statistical analyses were conducted using IBM SPSS Statistics for Windows, Version 24.0, 2016, Armonk, NY: IBM Corp.

## Results

The inclusion period was between 7 March 2016 and 10 December 2018, and the last 12-month follow-up was completed on 27 January 2020.

**Fig. 1:**
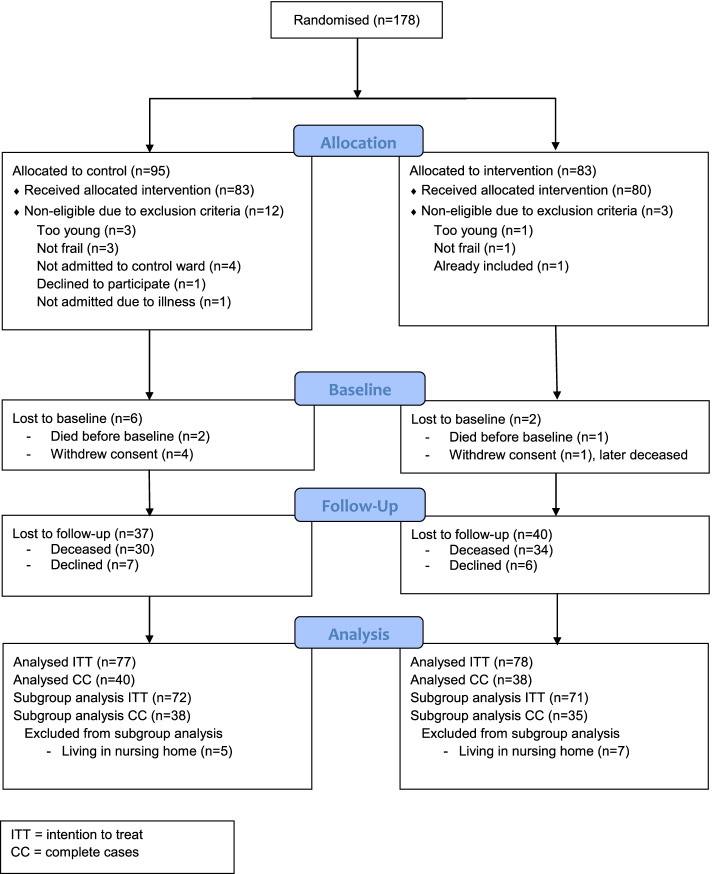
Flowchart of allocation, follow-ups, loss to follow-ups and analysis

As the hospital bed coordinators at the emergency department were responsible for the inclusion of participants but had no extra time for this task, they did not register all patients who declined to participate during the whole study period. However, based on the periods when this data was registered, we have estimated the number of eligible patients to be 210, of which 178 consented to participate (response rate: 85%) and received an allocation to control or intervention group [11]. Fifteen of these did not meet the inclusion criteria, and an additional eight were lost before the data collection began. Thus, 155 individuals participated in the study. The flowchart in Fig. 1 presents the details on the number of participants receiving allocated intervention at admission, reasons for declining participation at admission, the number of participants at the 12-month follow-up, and reasons for not participating at the follow-up.

As can be seen in Fig. 1, at admission 155 frail older people participated, 77 in the control group and 78 in the intervention group. At the 12-month follow-up, 78 participated, 40 in the control group and 38 in the intervention group. Mortality was the main reason for not participating at the follow-up.

The median age of the participants was 87 in the control group and 87.5 in the intervention group. Characteristics for the two groups are shown in Table 1.

**Table 1:** Characteristics of the participants in the two groups

	Control (*n* = 77)	Intervention (*n* = 78)	*p*-value
Age, mean (range)	86.2 (76–98)	87.5 (75–101)	0.17
Female % (n)	55.8 (43)	60.3 (47)	0.58
Living alone % (n)	62.3 (48)	65.4 (51)	0.70
Living in nursing home % (n)	6.5 (5)	9.0 (7)	0.27
Dependent in IADL % (n)	90.9 (70)	93.6 (73)	0.53
Dependent in PADL 2 weeks before admission % (n)	39.0 (30)	47.4 (37)	0.29
Dependent in PADL at baseline % (n)	68.8 (53)	76.9 (60)	0.26
Good self-rated health^a^% (n)	27.3 (21)	33.3 (26)	0.41
Non-frail % (n)	0	0	
Pre-frail % (n)	7.8 (6)	3.8 (3)	
Frail % (n)	92.2 (71)	96.2 (75)	0.33^2^
CIRS-G ≥ 3 in any category, % (n)^b^	93.5 (72)	98.7 (77)	0.26
CIRS-G, median number of ratings 3–4 (range)	3 (0–9)	3 (0–7)	

^a^ Excellent, very good, or good

^2^ Fischer’s exact test (too few numbers in one cell for chi-square)

^b^ Cumulative Illness Rating Scale for Geriatrics [28]. Rating 3 = severe/constant significant disability/uncontrollable chronic problem and rating 4 = extremely severe/immediate treatment required/end-organ failure/severe impairment in function

Reasons for admission are shown in Table 2. The most common reason was being admitted for dyspnoea or other difficulties with breathing, which could be due to for example pneumonia or heart failure. Having an impaired condition or an infection were also common reasons for admission.

**Table 2:** Reasons for admission

Reason for admission	Control (***n*** = 77)	Intervention (*n* = 78)	Total (***n*** = 155)
Dyspnoea/difficulty with breathing	30	21	51
Impaired condition/fatigue	11	18	29
Infection	12	17	29
Pathological blood count or status^a^	7	5	12
Abdominal pain	4	3	7
Chest pain	1	5	6
Syncope/absence attack	4	1	5
Vertigo	3	2	5
Swollen legs	1	3	4
Falls	3	1	4
Other^b^	1	2	3

^a^ For example anaemia, hyperglycaemia

^b^ Including dysphagia (1 intervention), head trauma (1 intervention), and thoracic pain (1 control)

As can be seen in Table 3, most participants were discharged directly back to their home. The participants in the intervention group were more likely to receive in-hospital geriatric rehabilitation before discharge, which was a statistically significant difference with OR = 4.93 and 95% CI = 1.35–18.08. They were also more likely to be discharged to a municipal short-stay nursing home, where they could receive care and rehabilitation (not statistically significant).

**Table 3:** Place of discharge after the hospital stay

Discharge to	Control (*n* = 77)	Intervention (*n* = 78)	Total (*n* = 155)
Home	59	41	100
In-hospital geriatric rehabilitation	3	13	16
Other hospital ward	4	4	8
Municipal short-stay nursing home	5	10	15
Back to nursing home	2	6	8
Deceased during hospital stay	4	3	7
Hospice	0	1	1

There were more participants in the intervention group who were less dependent in ADL compared to the control group from 2 weeks before hospital admission to the 12-month follow-up (statistically significant). This was true for all analyses: for intention-to-treat with imputated data for the attrition (OR = 3.3), for complete cases (OR = 3.7), and in the subgroups when those who lived in a nursing home were removed from the analysis (OR = 4.7 for intention to-treat analysis and OR = 5.3 for complete cases), see Table 4 and Supplementary Tables S1, S2, S3. There were also more participants in the intervention group who were less dependent in ADL from admission to the 12-month follow-up, but this difference was not as large, and only statistically significant in the analysis of complete cases.

**Table 4:** Change in ADL, self-rated health, and frailty to the 12-month follow-up, intention-to-treat analysis

	Control (*n* = 77)			Intervention (*n* = 78)				
	%	n	OR	%	n	OR	CI	*p*-value
ADL, change from 2 weeks before admission								
Improved	5.2	4	1	15.4	12	3.32	1.02–10.79	0.046
Maintained	19.5	15	1	14.1	11	0.68	0.29–1.59	0.37
Decreased	75.3	58	1	70.5	55	0.78	0.38–1.59	0.50
ADL, change from admission								
Improved	15.6	12	1	28.2	22	2.13	0.97–4.68	0.06
Maintained	14.3	11	1	14.1	11	0.99	0.40–2.43	0.97
Decreased	70.1	54	1	57.7	45	0.58	0.30–1.13	0.11
Self-rated health, change from admission								
Improved	16.9	13	1	19.2	15	1.17	0.52–2.66	0.70
Maintained	42.9	33	1	33.3	26	0.67	0.35–1.28	0.22
Decreased	40.3	31	1	47.4	37	1.34	0.71–2.53	0.37
Frailty, change from admission								
Improved	26.0	20	1	19.2	15	0.68	0.31–1.45	0.32
Maintained	23.4	18	1	20.5	16	0.85	0.39–1.81	0.67
Decreased	50.6	39	1	60.3	47	1.48	0.78–2.79	0.23

*OR* Odds ratio, *CI* 95% confidence interval.

There were no statistically significant changes from admission to the 12-month follow-up for self-rated health or frailty in any of the analyses, see Table 4 and Supplementary Tables S1, S2, S3.

Most of the participants who had improved in their ADL had improved in one activity. Two in the control group and three in the intervention group had improved in two activities, and two in the intervention group had improved in three activities. Table 5 shows the activities in which the participants had improved.

**Table 5:** ADL activities showing improvement. Number of participants

	ADL Activity	Control (***n*** = 4)	Intervention (***n*** = 12)	Total (***n*** = 16)
**IADL**	Cleaning	1	1	2
	Shopping	1	3	4
	Cooking	0	2	2
	Transportation	2	3	5
**PADL**	Bathing	0	1	1
	Dressing	2	1	3
	Going to the toilet	1	5	6
	Transferring	0	4	4
	Feeding	0	1	1

Of those who had improved in their ADL at the 12-month follow-up, five in the intervention group had received in-hospital geriatric rehabilitation and one had received rehabilitation in a municipal short-stay nursing home. All four in the control group who had improved in their ADL had been discharged to the home.

In addition, we analysed the ADL for different levels of frailty (5 or less frailty indicators or 6 or more indicators fulfilled) and for sex, with similar results in both groups and for both sexes (data not shown). The mortality at 1 year was 41.3% for the whole group, 39.0% in the control group and 43.6% in the intervention group (*p*-value 0.56).

## Discussion

The results from the randomised controlled study CGA-Swed show that CGA has positive effects on frail older people’s activities of daily living. The CGA decreased frail older people’s dependence in ADL to a higher extent than ordinary medical hospital care, with those receiving CGA and surviving up to 1 year after their hospital admission having up to 5 times higher odds for improvement. However, the study could not show any effect on self-rated health and frailty level.

Preventing dependence in ADL increases the ability to remain living in one’s own housing and not being forced to move to a nursing home [14]. Independence in daily activities is also strongly linked to older people’s health, well-being, and self-confidence [15, 29]. Thus, the fact that CGA can decrease frail older people’s dependence in ADL is very positive. This finding is not in accordance with the results of the Cochrane reviews, which could not find any significant effect on ADL dependence after receiving CGA [9, 10]. However, the reviews found that frail older people who had received CGA were more likely to be discharged back to their own housing. The discrepancy between the Cochrane reviews and the positive effects on ADL in our study could be due to different baselines when the ADL status was measured. The CGA-Swed study used 2 weeks before admission in addition to during admission, as this is more likely to estimate the habitual status of the person. Many other studies, however, may have used only ADL during admission to the hospital as their baseline; for example, most of the studies included in the Cochrane reviews measured ADL during admission [9, 10]. When acutely ill, there is often a decrease in ADL function, especially when the care provided does not consider the risk of this deterioration [5, 6]. CGA promotes early mobilisation in order to maintain as much independence in ADL as possible. Therefore, frail older persons who receive CGA might not lose as much in ADL as those who do not receive CGA. If the baseline is during admission, there might already be an effect of the CGA with less decline in ADL for those receiving CGA. This might make it more difficult to show positive effects of CGA if the comparison is made with the status during admission instead of their habitual status. In addition, the ADL status might change during the hospital stay, making the measurement sensitive to timing. Thus, we consider the use of frail older people’s habitual ADL status to be more accurate and less sensitive to bias.

The effect on ADL in the CGA-Swed study was more apparent after 12 months than after 1–6 months [16], which could be due to frail older people needing time to regain their functions in ADL. It could also be a survival effect where those who survive 1 year after the admission have the best development. The studies included in the Cochrane reviews that had follow-ups after 12 months showed more positive effects on ADL than those with shorter follow-ups [9, 10], which also indicates that frail older people might need longer to regain their ADL-function.

More participants in the intervention group received in-hospital geriatric rehabilitation and more were discharged to a municipal short-stay nursing home. This indicates that CGA acknowledges the need of rehabilitation to a higher extent, which probably is one explanation for the positive effects on ADL in the intervention group. However, many participants in both groups had decreased in their ADL at the 1 year follow-up. This, in addition to the high mortality, shows the vulnerability of this group of frail older people in need of acute hospital care.

The intervention group had somewhat higher mortality compared to the control group. However, this difference was not statistically significant, and was probably due to chance. In addition, the intention-to-treat analysis included imputation of worst case scenarios for the deceased, which makes the positive effect on ADL unlikely to be due to differences in mortality.

Since the hospital’s occupational therapy management decided not to prioritise patients in nursing homes for assessment by the occupational therapist, we did subgroup analyses without those living in nursing homes. This resulted in even higher odds for improved ADL after 1 year in the intervention group. This does not mean that frail older people living in nursing homes do not have any effects of receiving CGA – we had too few participants in nursing homes to be able to do further analyses on this subgroup.

The CGA-Swed study could not show any effect on self-rated health at the 12-month follow-up, or at the 1- and 6-month follow-ups [16]. ADL is known to influence self-rated health, but it is only one of many factors that influence perceived health [30]. This might explain why the positive effects on ADL after 1 year in this study were not accompanied by a similar effect on self-rated health.

Frailty is a dynamic process and natural remission is common among community-dwelling older people [31]. However, there were no effects on the development of frailty after one year in this RCT. Few studies have used frailty as an outcome of CGA in the acute care setting [32]. Dependence in ADL is a consequence of frailty rather than a part of frailty itself [2, 33], even though many frailty instruments include ADL items [34]. Thus, the intervention in the CGA-Swed study had an impact on the consequences of frailty in terms of affecting dependence in ADL, but the level of frailty itself was not affected. The reason might be that the participants had come so far in their development of frailty that further deterioration could not be prevented. Future research should examine larger samples and include more diverse levels of frailty to explore the effect the CGA has on different levels of frailty [2, 9, 10, 31, 35].

The study has several limitations. We nearly reached the estimated sample size, lacking only one participant. This made it possible to reach power for the primary outcome, but the sample was not large enough to conduct subgroup analyses regarding for example frailty level and disease burden. The power calculation was based on another study with frail older people seeking care at the emergency department [27]. As the mortality rate in the present study proved to be higher than in the other study, however, attrition was higher than expected. There is also a risk of mass significance, with results being statistically significant by chance. However, the positive effects on ADL were similar in all analyses, not only in a few. The participants are frail older people in need of acute hospital care, which is reflected in the characteristics of the participants at admission, with high levels of dependence in ADL and high morbidity [11]. The sample does not represent the general older population, and generalisability is limited to frail older people in need of acute in-hospital care. However, this is a vulnerable group with a high burden of disease and a high risk of further deterioration if their needs are not met, and we need more knowledge about this group. Since the sample did not include any participants who were not frail and very few who were pre-frail, we cannot compare the effects of CGA between frail, pre-frail and non-frail older people, and the sample size does not allow for subgroup analyses according to frailty level, which are further limitations [35]. The CGA-Swed study only included an intervention during the hospital stay and does not include any follow-ups in primary care or municipal care, which is a major limitation. A short intervention, lasting in many cases only a few days, has limited effect if it is not followed up after discharge. Better coordination and integration of care across different caregivers and care levels is important [5]. Further research on integrating CGA in the hospital with follow-ups in primary care and municipal care for frail older people is needed.

## Conclusions

The CGA-Swed study shows that frail older people are statistically significant more likely to improve in their ADL 1 year after discharge if they receive CGA during their hospital stay. Being less dependent in ADL increases frail older people’s ability to remain in their own housing, which is important for both the individual and society. It is important to support frail older people in continuing to be in charge of their daily activities of living even if they suffer from frailty and morbidity. More research is needed on the effects of CGA regarding different levels of frailty.

## Supplementary Information

**Additional file 1.**

## Data Availability

The dataset used and analysed in the current study is available from the corresponding author on reasonable request. De-identified participant data that underlie the results reported in this article will be available to the scientific community, immediately after publication and up to 5 years, upon request from researchers who provide a methodologically sound proposal and attempt to achieve aims in the approved proposal. Data will be stored for 10 years at the University of Gothenburg to enable review. Proposals should be directed to the corresponding author, katarina.wilhelmson@gu.se. To gain access, data requesters will need to sign a data access agreement. Data is covered by the Public Access to Information and Secrecy Act and a confidentiality assessment will be performed at each individual request. Permission from the University of Gothenburg, the Institute of Neuroscience and Physiology, has to be obtained before data can be accessed.

## Abbreviations

ADL: Activities of Daily Living
IADL: Instrumental Activities of Daily Living
PADL: Personal Activities of Daily Living
CGA: Comprehensive Geriatric Assessment
OR: Odds Ratio
CI: Confidence Interval
ITT: Intention To Treat
