# Supplementary material for: Positive effects on activities of daily living one year after receiving comprehensive geriatric assessment – results from the randomised controlled study CGA-Swed
Source: BMC Geriatr. 2022 Mar 3;22:180. doi: 10.1186/s12877-022-02862-6 (PMC8892729; doi:10.1186/s12877-022-02862-6)
Supplement: Supplementary file 1 — Additional file 1. [file 12877_2022_2862_MOESM1_ESM.docx]

# Supplementary Material

**Table S1. Change in ADL, self-rated health, and frailty to the 12-month follow-up, complete cases analysis.**

|  | Control (n=40) | | | Intervention (n=38) | | | | |
| --- | --- | --- | --- | --- | --- | --- | --- | --- |
|  | % | n | OR | % | n | OR | CI | p-value |
| ADL, change from 2 weeks before admission |  |  |  |  |  |  |  |  |
| Improved | 10.0 | 4 | 1 | 28.9 | 11 | 3.67 | 1.05–12.78 | 0.041 |
| Maintained | 37.5 | 15 | 1 | 23.7 | 9 | 0.52 | 0.19–1.38 | 0.19 |
| Decreased | 52.5 | 21 | 1 | 47.4 | 18 | 0.81 | 0.33–1.98 | 0.65 |
|  |  |  |  |  |  |  |  |  |
| ADL, change from admission |  |  |  |  |  |  |  |  |
| Improved | 30.0 | 12 | 1 | 55.3 | 21 | 2.88 | 1.14–7.13 | 0.026 |
| Maintained | 25.0 | 10 | 1 | 15.8 | 6 | 0.56 | 0.18–1.74 | 0.32 |
| Decreased | 45.0 | 18 | 1 | 28.9 | 11 | 0.50 | 0.19–1.27 | 0.14 |
|  |  |  |  |  |  |  |  |  |
| Self-rated health, change from admission |  |  |  |  |  |  |  |  |
| Improved | 32.5 | 13 | 1 | 39.5 | 15 | 1.35 | 0.54–3.43 | 0.52 |
| Maintained | 45.0 | 18 | 1 | 34.2 | 13 | 0.64 | 0.25–1.59 | 0.33 |
| Decreased | 22.5 | 9 | 1 | 26.3 | 10 | 1.23 | 0.44–3.46 | 0.70 |
|  |  |  |  |  |  |  |  |  |
| Frailty, change from admisssion^1^ |  | 35 |  |  | 34 |  |  |  |
| Improved | 54.3 | 19 | 1 | 41.2 | 14 | 0.59 | 0.23–1.53 | 0.28 |
| Maintained | 31.4 | 11 | 1 | 32.4 | 11 | 1.04 | 0.38–2.87 | 0.93 |
| Decreased | 14.3 | 5 | 1 | 26.5 | 9 | 2.16 | 0.64–7.28 | 0.21 |

^1^ Information missing for frailty indicators requiring physical measurements for an additional 9 participants at the 12-month follow-up (intervention 4, control 5) due to the interviews being performed by telephone or in one case the participant refusing further questions/measurements.

OR: Odds ratio; CI: 95% confidence interval

**Table S2. Change in ADL, self-rated health, and frailty to the 12-month follow-up. Subgroup analysis, intention to treat, without those living in nursing homes.**

|  | Control (n=72) | | | Intervention (n=71) | | | | |
| --- | --- | --- | --- | --- | --- | --- | --- | --- |
|  | % | n | OR | % | n | OR | CI | p-value |
| ADL, change from 2 weeks before admission |  |  |  |  |  |  |  |  |
| Improved | 4.2 | 3 | 1 | 16.9 | 12 | 4.68 | 1.26–17.37 | 0.021 |
| Maintained | 20.8 | 15 | 1 | 12.7 | 9 | 0.55 | 0.22–1.36 | 0.20 |
| Decreased | 75.0 | 54 | 1 | 70.4 | 50 | 0.79 | 0.38–1.66 | 0.54 |
|  |  |  |  |  |  |  |  |  |
| ADL, change from admission |  |  |  |  |  |  |  |  |
| Improved | 15.3 | 11 | 1 | 28.2 | 20 | 2.17 | 0.95–4.96 | 0.065 |
| Maintained | 15.3 | 11 | 1 | 14.1 | 10 | 0.91 | 0.36–2.30 | 0.84 |
| Decreased | 69.4 | 50 | 1 | 57.7 | 41 | 0.60 | 0.30–1.20 | 0.15 |
|  |  |  |  |  |  |  |  |  |
| Self-rated health, change from admission^1^ |  | 71 |  |  | 70 |  |  |  |
| Improved | 15.5 | 11 | 1 | 18.6 | 13 | 1.24 | 0.52–3.00 | 0.63 |
| Maintained | 42.2 | 30 | 1 | 34.3 | 24 | 0.71 | 0.36–1.41 | 0.33 |
| Decreased | 42.2 | 30 | 1 | 47.1 | 33 | 1.22 | 0.63–2.37 | 0.56 |
|  |  |  |  |  |  |  |  |  |
| Frailty, change from admission |  |  |  |  |  |  |  |  |
| Improved | 26.4 | 19 | 1 | 19.7 | 14 | 0.69 | 0.31–1.50 | 0.34 |
| Maintained | 22.2 | 16 | 1 | 18.3 | 13 | 0.78 | 0.35–1.78 | 0.56 |
| Decreased | 51.4 | 37 | 1 | 62.0 | 44 | 1.54 | 0.79–3.00 | 0.20 |

^1^One in control group and one in intervention group missing at baseline.

OR: Odds ratio; CI: 95% confidence interval

**Table S3. Change in ADL, self-rated health, and frailty to the 12-month follow-up. Subgroup analysis, complete cases, without those living in nursing homes.**

|  | Control (n=38) | | | Intervention (n=35) | | | | |
| --- | --- | --- | --- | --- | --- | --- | --- | --- |
|  | % | n | OR | % | n | OR | CI | p-value |
| ADL, change from 2 weeks before admission |  |  |  |  |  |  |  |  |
| Improved | 7.9 | 3 | 1 | 31.4 | 11 | 5.35 | 1.35–21.22 | 0.017 |
| Maintained | 39.5 | 15 | 1 | 22.9 | 8 | 0.45 | 0.16–1.26 | 0.13 |
| Decreased | 52.6 | 20 | 1 | 45.7 | 16 | 0.76 | 0.30–1.90 | 0.56 |
|  |  |  |  |  |  |  |  |  |
| ADL, change from admission |  |  |  |  |  |  |  |  |
| Improved | 28.9 | 11 | 1 | 54.3 | 19 | 2.91 | 1.11–7.66 | 0.030 |
| Maintained | 26.3 | 10 | 1 | 17.1 | 6 | 0.58 | 0.19–1.81 | 0.35 |
| Decreased | 44.7 | 17 | 1 | 28.6 | 10 | 0.49 | 0.19–1.31 | 0.16 |
|  |  |  |  |  |  |  |  |  |
| Self-rated health, change from admisssion^1^ |  | 37 |  |  |  |  |  |  |
| Improved | 29.7 | 11 | 1 | 37.1 | 13 | 1.40 | 0.52–3.73 | 0.50 |
| Maintained | 48.6 | 18 | 1 | 34.3 | 12 | 0.55 | 0.21–1.42 | 0.22 |
| Decreased | 21.6 | 8 | 1 | 28.6 | 10 | 1.45 | 0.50–4.24 | 0.50 |
|  |  |  |  |  |  |  |  |  |
| Frailty, change from admission^2^ |  | 33 |  |  | 31 |  |  |  |
| Improved | 54.5 | 18 | 1 | 41.9 | 13 | 0.60 | 0.22–1.62 | 0.31 |
| Maintained | 30.3 | 10 | 1 | 29.0 | 9 | 0.94 | 0.32–2.75 | 0.91 |
| Decreased | 15.2 | 5 | 1 | 29.0 | 9 | 2.29 | 0.67–7.82 | 0.19 |

^1^ One in control group missing at baseline.

^2^ Information missing for frailty indicators requiring physical measurements for an additional 9 participants at the 12-month follow-up (intervention 4, control 5) due to the interviews being performed by telephone or in one case the participant refusing further questions/measurements.

OR: Odds ratio; CI: 95% confidence interval
